# Estimated Global Mortality Attributable to Smoke from Landscape Fires

**DOI:** 10.1289/ehp.1104422

**Published:** 2012-02-18

**Authors:** Fay H. Johnston, Sarah B. Henderson, Yang Chen, James T. Randerson, Miriam Marlier, Ruth S. DeFries, Patrick Kinney, David M.J.S. Bowman, Michael Brauer

**Affiliations:** 1 Menzies Research Institute, University of Tasmania, Hobart, Tasmania, Australia; 2 University of Tasmania, Hobart, Tasmania, Australia; 3 British Columbia Centre for Disease Control, Vancouver, British Columbia, Canada; 4 School of Population and Public Health, University of British Columbia, Vancouver, British Columbia, Canada; 5 Department of Earth System Science, University of California–Irvine, Irvine, California, USA; 6 Department of Earth and Environmental Sciences,; 7 Ecology, Evolution, and Environmental Biology, and; 8 Mailman School of Public Health, Columbia University, New York, New York, USA; 9 School of Plant Science, University of Tasmania, Hobart, Tasmania, Australia

**Keywords:** air pollution, biomass burning, carbon cycle, deforestation, global burden of disease, landscape fire smoke, mortality

## Abstract

Background: Forest, grass, and peat fires release approximately 2 petagrams of carbon into the atmosphere each year, influencing weather, climate, and air quality.

Objective: We estimated the annual global mortality attributable to landscape fire smoke (LFS).

Methods: Daily and annual exposure to particulate matter ≤ 2.5 μm in aerodynamic diameter (PM_2.5_) from fire emissions was estimated globally for 1997 through 2006 by combining outputs from a chemical transport model with satellite-based observations of aerosol optical depth. In World Health Organization (WHO) subregions classified as sporadically affected, the daily burden of mortality was estimated using previously published concentration–response coefficients for the association between short-term elevations in PM_2.5_ from LFS (contrasted with 0 μg/m^3^ from LFS) and all-cause mortality. In subregions classified as chronically affected, the annual burden of mortality was estimated using the American Cancer Society study coefficient for the association between long-term PM_2.5_ exposure and all-cause mortality. The annual average PM_2.5_ estimates were contrasted with theoretical minimum (counterfactual) concentrations in each chronically affected subregion. Sensitivity of mortality estimates to different exposure assessments, counterfactual estimates, and concentration–response functions was evaluated. Strong La Niña and El Niño years were compared to assess the influence of interannual climatic variability.

Results: Our principal estimate for the average mortality attributable to LFS exposure was 339,000 deaths annually. In sensitivity analyses the interquartile range of all tested estimates was 260,000–600,000. The regions most affected were sub-Saharan Africa (157,000) and Southeast Asia (110,000). Estimated annual mortality during La Niña was 262,000, compared with 532,000 during El Niño.

Conclusions: Fire emissions are an important contributor to global mortality. Adverse health outcomes associated with LFS could be substantially reduced by curtailing burning of tropical rainforests, which rarely burn naturally. The large estimated influence of El Niño suggests a relationship between climate and the burden of mortality attributable to LFS.

Landscape fires (encompassing wild and prescribed forest fires, tropical deforestation fires, peat fires, agricultural burning, and grass fires) release approximately 2 petagrams (2 × 10^12^ kg) of carbon into the atmosphere annually (van der Werf et al. 2010). These emissions affect planetary processes such as radiative forcing (which influences average global temperatures) and hydrological cycles (which influence regional cloud formation and rainfall) (Bowman et al. 2009; Cochrane and Laurance 2008; Fargione et al. 2008; Langmann et al. 2009; Tosca et al. 2010; Yokelson et al. 2007). Most emissions originate from fires set in tropical rainforests and savannas, where they cause recurrent episodes of severe pollution that affect some of the poorest regions of the world (van der Werf et al. 2010). Despite extensive literature describing the harmful effects of air pollution, the health impacts of landscape fire smoke (LFS) are rarely highlighted in discussions about fires and their role in the earth system (Lohman et al. 2007).

Smoke from the combustion of biomass is composed of hundreds of chemicals, many of which are known to be harmful to human health (Naeher et al. 2007). The most important risk-related measure of smoke is particulate matter (PM) with an aerodynamic diameter ≤ 2.5 μm (PM_2.5_). This PM primarily consists of organic carbon and black carbon components, along with smaller contributions from inorganic species (Naeher et al. 2007; Reid et al. 2005). PM is also produced by the combustion of fossil fuels, and most health evidence for PM_2.5_ comes from studies in urban environments (Pope and Dockery 2006). Urban PM has been associated with a wide range of adverse health outcomes including all-cause, neonatal and cardiorespiratory mortality, exacerbations of respiratory and cardiovascular conditions, and pathophysiological changes such as inflammation, oxidative stress, and procoagulation (Pope and Dockery 2006). The effects of PM derived from burning biomass have been less extensively investigated, and much of the evidence comes from studies of air pollution from household solid fuel use (Naeher et al. 2007). A handful of toxicological studies suggest that biomass smoke particles elicit pathophysiological effects similar to those of urban PM (Barregard et al. 2006; Danielsen et al. 2009; Kocbach et al. 2008). Although there are relatively few epidemiological studies on smoke-related PM, they also report outcomes consistent with those elicited by urban PM, including increased all-cause mortality and exacerbations of respiratory conditions (Delfino et al. 2009; Hänninen et al. 2009; Johnston et al. 2007, 2011; Morgan et al. 2010; Sastry 2002). However, evidence concerning cardiovascular outcomes of smoke-related PM remains scarce and inconclusive (Naeher et al. 2007; Sanhueza et al. 2009). Results from several studies of the extensive rainforest and peat fires in Southeast Asia in 1997 through 1998 suggest substantial health and economic impacts of LFS (Jayachandran 2009; Mott et al. 2005; Sastry 2002; Schweithelm et al. 2006). Further, fires are becoming more widespread and frequent in some regions (Turetsky et al. 2011; Westerling et al. 2006), and this source of air pollution is likely to continue to grow in magnitude and consequent health impacts (Confalonieri et al. 2007; Denman et al. 2007; Langmann et al. 2009). Because fire emissions contribute to radiative forcing, there is potential for the development of a positive feedback between a warming climate and increasingly severe fire events in several biomes (Bowman et al. 2009). In this context, a global assessment of the mortality impacts of LFS is required.

## Materials and Methods

Studying the magnitude of health impacts from LFS presents several technical challenges, including estimation of the exposure to smoke-specific PM for each spatial unit of analysis, selection of the most appropriate concentration–response functions, and consideration of what theoretic minimum (counterfactual) exposure values to apply. Moderate to high levels of uncertainty are associated with many of these steps, so our objectives were to provide a reasonable principal estimate given the available data and then to evaluate the sensitivity of the principal estimate to the assumptions used in the principal analysis. The World Health Organization (WHO) Global Burden of Disease (GBD) Comparative Risk Assessment framework provides a standard set of methods for this and has previously been used to evaluate the annual mortality attributable to urban air pollution and to indoor air pollution from household solid fuel use (Ezzati et al. 2002; Lopez et al. 2006a). Methods for estimating the global mortality associated with particulate air pollution are being revised in the light of new epidemiological evidence and exposure assessment methods, and new cause-specific results are expected in 2012 (Institute for Health Metrics and Evaluation 2010). However, the epidemiological evidence concerning LFS remains limited, and evidence concerning LFS and cause-specific mortality is not currently available. For this reason, our analyses evaluate all-cause mortality.

**Figure 1:**
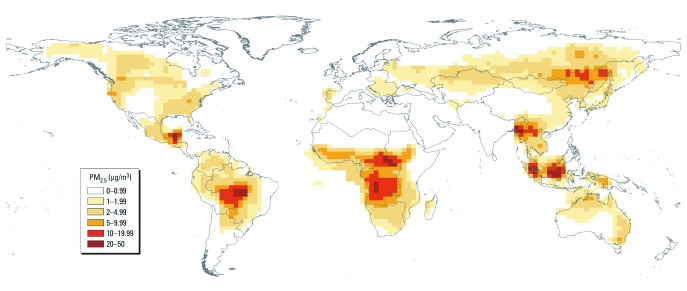
Estimated annual average (1997–2006) PM_2.5_ concentrations from landscape fires, combining estimates from the GEOS-Chem model with the MODIS and MISR optimizations.

**Figure 2:**
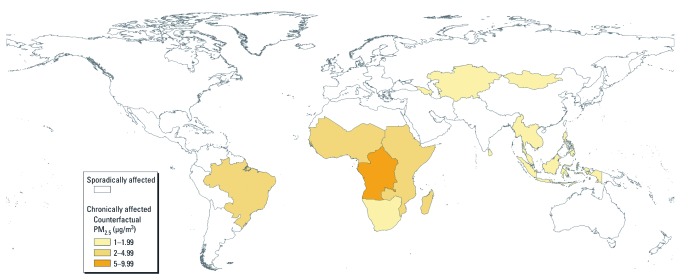
WHO subregions classified as sporadically and chronically affected. Subregions were classified as chronically affected if ≥ 50% of their populations and/or ≥ 50% of their land areas were covered by smoke-affected exposure cells for at least 3 months per year for ≥ 5 years. The theoretical minimum annual average (counterfactual) concentration used for chronically affected subregions was calculated by taking the mean of the minimum 12-month running average (over 120 months) of all exposure cells in the subregion. The remaining subregions were classified as sporadically affected. The theoretical minimum daily average (counterfactual) concentration used for sporadically affected subregions was zero.

*Input data.* Exposure estimates. We combined information from satellite-derived observations of global fire activity, geographic area burned, and type of vegetation burned in a global atmospheric three-dimensional (3-D) chemical transport model. We then combined output from that model with satellite-based measurements of aerosol optical depth (AOD) to estimate annual PM_2.5_ emissions from landscape fires. For a detailed description of the exposure estimates, see Supplemental Material, pp. 3–8, Table 1, and Figures 1 and 2 (http://dx.doi.org/10.1289/ehp.1104422). A summary is presented below.

**Table 1:** Estimates of the global and regional annual mortality attributable to LFS and estimates from 2 years that corresponded with strong El Niño and La Niña conditions.

Scenario	Global	Sub-Saharan Africa>^a^	Southeast Asia^b^	South America^c^
Annual average (1997–2006)		339,000		157,000		110,000		10,000
EL Niño year (September 1997–August 1998)		532,000		137,000		296,000		19,000
La Niña year (September 1999–August 2000)		262,000		157,000		43,000		11,000
Results are shown for the three most severely smoke-affected regions. These estimates are based on the assumptions used in the principal analysis (see Table 2). aWHO subregions 18–21. bWHO subregion 5 only. cWHO subregions 11–14.								

Monthly resolved emissions estimates were obtained from the Global Fire Emission Database (Global Fire Data 2012; van der Werf et al. 2006), which combines satellite observations of burned area (in square kilometers) with estimates of fuel loads obtained from a biogeochemical model (Giglio et al. 2006). These emissions estimates were used in the GEOS-Chem global 3-D chemical transport model (Bey et al. 2001), which simulates the transport, transformation, and deposition of organic carbon and black carbon aerosols. The model had a 2° (latitude) × 2.5° (longitude) horizontal resolution ~ 222 × 278 km at the equator and 30 vertical layers (Bey et al. 2001). We performed two sets of GEOS-Chem simulations spanning a 10-year period (1997 through 2006). The first included all aerosol emission sources (fossil fuel, biofuel, landscape fires, natural dust, and sea salt), whereas the second excluded landscape fire emissions to separate the contribution from this source.

Finally, we scaled the modeled PM_2.5_ estimates using two sets of AOD observations from the Moderate Resolution Imaging Spectroradiometer (MODIS) and the Multiangle Imaging Spectroradiometer (MISR) aboard the U.S. National Aeronautics and Space Administration (NASA) *Terra* satellite (Martonchik et al. 2009; Remer et al. 2005). We maintained the same seasonal, regional, and vertical aerosol distributions as predicted by the GEOS-Chem simulations. Our best estimate of surface PM_2.5_ (1997 through 2006 average shown in Figure 1) combined information from the model estimates along with the two satellite AOD-scaled estimates:

LFS PM_2.5_ = [(2 × MODEL) + MODIS + MISR]/4, [1]

where MODEL is the estimate of PM_2.5_ from LFS derived from GEOS-Chem and MODIS and MISR are the two satellite AOD-scaled estimates. We multiplied the model contribution by 2 so that our best estimate gave equal weight to the *a priori* atmospheric model distribution and the sum of the two satellite-scaled estimates. The total aerosol emissions from fires used in the model simulations was 23.5 teragrams (Tg; 1 Tg = 10^9^ kg) per year averaged over 1997 through 2006. Comparable estimates for the MISR and MODIS AOD-based optimizations were 55.0 and 45.5 Tg/year, respectively [see Supplemental Material, Table 1 (http://dx.doi.org/10.1289/ehp.1104422)] and were within the range of previously published estimates (see Supplemental Material, Table 2). Our best estimate, defined according to Equation 1, was 36.9 Tg/year.

**Table 2:** Results of sensitivity analyses indicating the influence of varying individual assumptions on annual global mortality estimates: proportion of principal estimate of annual mortality, when all other principal analysis assumptions are held constant.

Source of uncertainty/principal analysis assumption and variations	Annual mortality proportion
Estimated PM2.5 concentrations	
Principal analysis: LFS PM2.5 concentrations estimated from the combination of a global chemical transport model GEOS-Chem and satellite-derived aerosol data from MODIS and MISR	1.00
MODEL: PM2.5 concentrations estimated from the GEOS-Chem global chemical transport model	0.68
MODIS: MODEL estimate optimized using satellite-derived aerosol data from MODIS	1.47
MISR: MODEL estimates optimized using satellite-derived aerosol data from MISR	1.20
Pattern of exposure	
Principal analysis: mortality in sporadically affected subregions estimated using daily average exposure estimates and response functions; mortality in chronically affected WHO subregions estimated using yearly mean exposure estimates and response functions	1.00
Sporadic only: mortality in all subregions estimated using daily average exposure estimates and response functions	0.41
Chronic only: mortality in all subregions estimated using yearly average exposure estimates and response functions	1.54
Shape of concentration–response function	
Principal analysis: mortality response calculated as a linear function of the PM2.5 concentration	1.00
Log-linear: mortality response calculated as a function of the logarithm of the PM2.5 concentration	2.31
Counterfactual exposure estimates for chronically affected regions	
Principal analysis: the counterfactual estimated for each WHO subregion as the mean of the minimum 12-month running-average smoke-specific PM2.5 concentration for each exposure cell within the subregion	1.00
Zero: a global value of 0 μg/m3	1.44
La Niña: cell-by-cell average for a La Niña year, September 1999–August 2000 inclusive	0.45
La Niña regional average: regional average of the values from La Niña	0.81
Cell-by-cell minimum: minimum of the 12-month running averages of each cell	0.78
Cell-by-cell categorization: global categorization of the values above at the 90th, 97th, and 99th percentiles, applying the average of the category to all cells in the category	0.82
Maximum yearly average exposure used for estimating chronic mortality impacts	
Principal analysis: maximum exposure of 50 μg/m3 was used for estimating the mortality associated with chronic exposure	1.00
Maximum exposure of 30 μg/m3 was used for estimating the mortality associated with chronic exposure	0.99
Range of minimum and maximum daily exposures used for estimating sporadic exposure impacts	
Principal analysis: range of exposure assessed was 5–200 μg/m3	1.00
Most restrictive range tested: 10–100 μg/m3	0.98
Least restrictive range tested: 1–300 μg/m3	1.01

Evaluation of exposure estimates. Surface measurements of PM_2.5_ are not available for most regions with high fire emissions. To evaluate the quality of the global exposure estimates, we used ground-based AOD from National Aeronautics and Space Administration’s (NASA) Aerosol Robotic Network (AERONET; NASA 2012) (Holben et al. 1998), PM_2.5_ measurements from the U.S. Environmental Protection Agency IMPROVE (Interagency Monitoring of Protected Visual Environments) program (Chow and Watson 2002), and visibility data in tropical regions from the National Climatic Data Center Global Summary of the Day (National Oceanic and Atmospheric Administration 2009). Our exposure estimates correlated well with these other measures in regions with high fire activity [see Supplemental Material, Figures 3–6 (http://dx.doi.org/10.1289/ehp.1104422)]. Correlations (Pearson’s *r*) of estimated AOD with monthly mean AODs from AERONET were 0.81 in southern Africa (*n* = 119), 0.90 in northern Africa (*n* = 74), and 0.76 in Southeast Asia (*n* = 148; see Supplemental Material, Figure 4). Median correlations between PM_2.5_ and visibility were 0.57 for sub-Saharan Africa (*n* = 58), 0.60 for South America (*n* = 47), and 0.68 for Southeast Asia (*n* = 13; see Supplemental Material, Figure 6).

**Figure 3:**
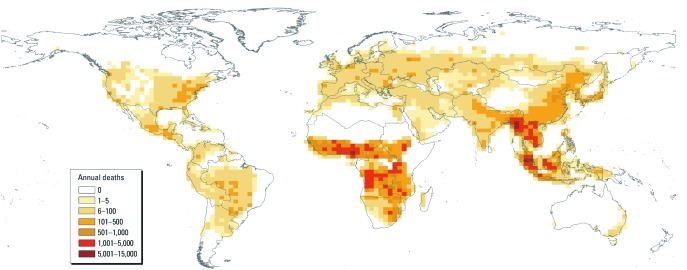
Map showing the principal estimates of the annual average (1997–2006) global mortality attributable to LFS.

**Figure 4:**
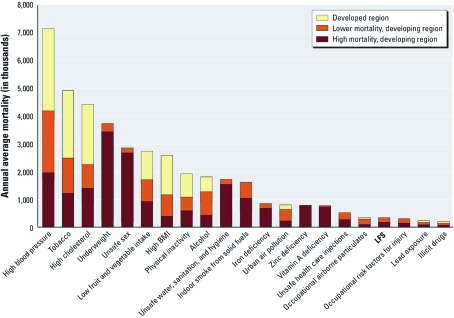
Annual mortality estimate for LFS in the context of estimates for other modifiable risk factors assessed as part of the WHO GBD studies (adapted from Ezzati et al. 2002).

Gridded mortality estimates. Country-specific estimates for all-cause all-age mortality in the year 2002 were obtained from the WHO Global Health Observatory (2011). Estimates from the Gridded Population of the World (GPW; version 3) project were used to map country-specific mortality onto the 2° × 2.5° exposure cells (Sociodemographic Data and Applications Centre 2011). The spatial resolution of the GPW data is 2.5 arc-min (~ 4.6 × 4.6 km at the equator), meaning that each exposure cell encompassed 2,880 population cells. To distribute mortality between the population cells, we assigned each cell to the underlying country that contained most of it, summed the GPW population for each country and calculated the percentage of the total population in each cell, and then assigned that percentage of the national mortality to the cell. In the < 1% of cases where population cells were assigned to countries that do not belong to the WHO, we followed the same steps for the 21 WHO subregions and assigned those values instead. The mortality in each exposure cell was estimated by summing the mortality in the 2,880 underlying population cells.

*Global burden calculations.* Pattern of exposure: subregions of sporadic and chronic impact. Fire activity varied widely across the globe during the 1997 through 2006 period. Some areas were affected sporadically, with a limited number of smoky days in any given year; some areas were affected chronically, with whole seasons being smoke-affected in multiple years. Our principal analysis treats these areas as fundamentally different because acute and chronic PM exposures have independent health effects (Pope and Dockery 2006; Schwartz 2000). We began by classifying each of the 21 WHO subregions as sporadically affected or chronically affected.

The complete set of smoke-specific PM_2.5_ estimates (12 months × 10 years × 4,208 exposure cells = 504,960) was log-normally distributed with a 90th percentile value of 3 μg/m^3^. When concentration estimates were rounded to integers, most exposure cells had a value of zero in most months (331,035 of 504,960), indicating low smoke-specific PM_2.5_. An exposure cell with a 1-month smoke-specific PM_2.5_ estimate > 3 μg/m^3^ was classified as being smoke affected during that month. Exposure cells with ≥ 3 smoke-affected months in ≥ 5 of the years were classified as chronically affected (732 of 4,208). Exposure cells that were not chronically affected were classified as sporadically affected (3,476 of 4,208). A WHO subregion was classified as chronically affected if > 50% of its population and/or > 50% of its land area was covered by chronically affected exposure cells (7 of 21; Figure 2). All other WHO regions were classified as sporadically affected (14 of 21; Figure 2).

Burden for sporadically affected subregions. For sporadically affected subregions, we estimated effects of short-term (daily) fluctuations in smoke-specific PM_2.5_ concentrations on mortality. Daily output from GEOS-Chem was used to estimate the number of days per year that PM_2.5_ concentrations exceeded a set of threshold values (300, 200, 100, 50, 40, 30, 20, 10, 5, 4, 3, 2, and 1 μg/m^3^). These threshold values were chosen to provide a range of possible concentrations for sensitivity analyses, and because they reflect clinically relevant increments (10 μg/m^3^, 100 μg/m^3^) reported in the literature.

The annual mortality attributable to LFS in each sporadically affected 2° × 2.5° exposure cell was calculated as

Sporadically affected attributable mortality =


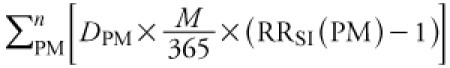
[2]

where PM is one smoke-specific PM_2.5_ threshold concentration out of *n* possible threshold values (see above), *D*_PM_ is the number of days between PM and the next highest concentration, *M* is the annual number of deaths in the exposure cell, and RR_SI_ is a relative rate estimate for all-cause mortality due to short-term PM exposure. Although annual mortality is not evenly distributed among the 365 days of the year, there are insufficient data to estimate seasonal mortality on a global scale.

For the principal analysis, a linear RR_SI_ estimate of 0.11% [95% confidence interval (CI): 0, 0.26%] per increase of 1 μg/m^3^ was used with minimum and maximum concentrations of 5 and 200 μg/m^3^. This means that cells with daily exposure estimates of < 5 μg/m^3^ were not included, and cells with exposure estimates > 200 μg/m^3^ were fixed at a value of 200 μg/m^3^. The RR_SI_ was calculated using the average (weighted by the inverse of the standard errors) of values from studies reporting associations between all-cause mortality and short-term elevations of ambient PM_10_ during fire events (Morgan et al. 2010; Sastry 2002) and PM_2.5_ (Hänninen et al. 2009). Associations with ambient PM_10_ were converted to associations with PM_2.5_ by assuming that 75% of all particles < 10 μm were also < 2.5 μm. This is halfway between the 90% ratio measured during fire events (Ward and Hardy 1991) and the 60% ratio used by Cohen et al. (2004) in the initial GBD estimate for urban air pollution.

Burden for chronically affected subregions. No studies have yet reported on the mortality impacts of long-term exposure to LFS. As such, we estimated all-cause mortality in chronically affected exposure cells by assuming the effects of smoke-related PM to be the same as those of urban PM. Specifically, for the principal analysis we assumed a linear 0.64% (95% CI: 0.35%, 0.94%) increase in annual all-cause mortality for each 1-μg/m^3^ increase in the long-term smoke-specific PM_2.5_ average, as reported in the American Cancer Society study on urban air pollution (Pope et al. 1995). This is one of the most conservative concentration–response estimates that has been reported in multiple studies of urban PM (Pope and Dockery 2006). The maximum concentration of effect was assumed to be 50 μg/m^3^. This means that cells with annual exposure estimates > 50 μg/m^3^ were fixed at a value of 50 μg/m^3^. The annual mortality attributable to LFS in each chronically affected exposure cell was calculated as

Chronically affected attributable mortality = *M* × ([RR_CI_(PM – CF)] – 1), [3]

where PM is the estimated average annual smoke-specific PM_2.5_ concentration in the exposure cell based on estimates for 1997 through 2006, CF is the counterfactual concentration for the WHO subregion in which the exposure cell was located, *M* is the annual number of deaths in the exposure cell, and RR_CI_ is the relative rate of all-cause mortality for long-term PM exposure (i.e., 0.64% for the principal analysis).

The counterfactual concentration is the theoretical minimum annual smoke-specific PM_2.5_ concentration under ideal conditions. For example, if landscape fires were completely eliminated worldwide, the global counterfactual value would be zero. Given that fire is a natural part of the earth system, we used a more data-driven approach to set counterfactual values for chronically affected WHO subregions. We used a subregion-wide approach because emissions from similar landscapes in neighboring countries can vary widely because of different land management practices, so the theoretical minimum exposure estimated for a single exposure cell might not truly reflect the minimum exposure possible for that particular landscape (Bowman et al. 2011). Specifically, we determined the smallest 12-month running average smoke-specific PM_2.5_ concentration for each exposure cell within a WHO subregion, and averaged the minimum annual concentrations across all exposure cells to determine the counterfactual value for that WHO subregion.

*Sensitivity analyses.* There are several sources of uncertainty in our inputs, and we addressed these through multiple sensitivity analyses. First, we assumed both linear and log-linear forms for the concentration–response functions (i.e., RR_SI_ in Equation 2 and RR_CI_ in Equation 3). Although there is increasing evidence of a log-linear association for cardiovascular mortality related to urban air pollution (Pope et al. 2011), we used the linear assumption for the principal analysis because studies on the cardiovascular effects of LFS have been inconclusive. We also tested a range of different exposure limits. For the sporadic assumption, the minimum concentration was varied between 1 and 10 μg/m^3^ and the maximum was varied between 50 and 300 μg/m^3^. For the chronic assumption, five alternative counterfactual definitions [a global value of 0 μg/m^3^; cell-by-cell average for a La Niña year, September 1999–August 2000 inclusive; regional average of the values from La Niña; minimum of the 12-month running averages of each cell; and global categorization of the values above at the 90th, 97th, and 99th percentiles, applying the average of the category to all cells in the category] were tested with maximum yearly average concentrations at 30 and 50 μg/m^3^. We repeated analyses using the GEOS-Chem and satellite AOD-scaled exposure estimates separately. To assess the effect of our assumptions concerning the combination of sporadic and chronic exposures, all analyses were repeated with all subregions classified as being sporadically affected and with all subregions being classified as chronically affected. There is large interannual variation in emissions from landscape fires mostly driven by changes in climatic conditions (van der Werf et al. 2008). To assess the influence of interannual climatic variability, analyses were repeated with concentration estimates for a strong El Niño year that occurred between September 1997 and August 1998 (inclusive) and a strong La Niña year that occurred between September 1999 and August 2000 (inclusive) (van der Werf et al. 2004).

## Results

*Exposure.* Estimated annual average concentrations ranged from 0 to 45 μg/m^3^ annually (mean = 1.8 μg/m^3^; Figure 1). The population-weighted annual average was 2.1 μg/m^3^, ranging from 0.2 μg/m^3^ in the Caribbean subregion to 12.2 μg/m^3^ in sub-Saharan Africa. The population-weighted average number of annual days > 5 μg/m^3^ was 28, ranging from 6 in the Caribbean subregion to 141 in sub-Saharan Africa.

*Burden of mortality.* Our principal estimate for the average annual mortality associated with exposure to LFS was 339,000 worldwide, including 157,000 in sub-Saharan Africa and 110,000 in Southeast Asia (Figure 3). The estimates for mortality due to LFS exposure compared with no LFS exposure at all (i.e., a zero exposure counterfactual) were 286,000 in sub-Saharan Africa and 119,000 in Southeast Asia, reflecting much higher background fire activity in sub-Saharan Africa than in Southeast Asia. During the El Niño year, the estimated mortality was higher, particularly in Southeast Asia, where El Niño is associated with dry conditions and more fires (Table 1).

Outputs from all tested models (*n* = 2,192) had a median of 379,000 and interquartile range of 260,000–600,000 [see Supplemental Material, Figure 7 (http://dx.doi.org/10.1289/ehp.1104422)]. Results of the sensitivity analyses are shown in Table 2. If a log-linear, rather than linear, concentration–response function was assumed, the mortality estimates more than doubled. The results were also sensitive to the exposure estimates, the assumed pattern of exposure (sporadic vs. chronic), and the choice of the counterfactual exposure estimation, all of which caused the estimated mortality to vary between 0.41 and 1.54 times the principal estimate (Table 2). Results were minimally influenced by the maximum and minimum exposures of effect, which caused the estimates to vary just 0.98 to 1.01 times the principal estimate (Table 2).

## Discussion

Our estimate of 339,000 annual deaths attributable to exposure to LFS is lower than estimates for urban air pollution (800,000) and much lower than estimates for household solid fuel use (1,600,000) (Lopez et al. 2006b). Similar to other environmental risk factors such as unsafe water and indoor and urban air pollution, the mortality burden attributable to LFS falls disproportionately on low-income regions of the world (Figure 4) (Ezzati et al. 2002).

The major strengths of these analyses lie in the use of existing global data sets for terrestrial fire emissions, meteorology, population density, and mortality. Using the WHO geographic subregions and mortality estimates helped make our findings comparable with previously reported estimates for other environmental risk factors. However, there are many limitations inherent in compiling and modeling data at a global scale. A major source of uncertainty comes from the emission factors for fire-derived aerosols that were used to model the exposure estimates. We used emission factors at the lower end of the range in the literature [see Supplemental Material, Table 2 (http://dx.doi.org/10.1289/ehp.1104422)] even though larger emission factors have been shown to improve model estimates of PM_2.5_ compared with satellite and surface network observations (Chin et al. 2009; Reid et al. 2009). In addition, the sum of the black carbon and organic carbon emissions factors was often lower than the observed PM_2.5_ emissions factors, likely resulting in GEOS-Chem underestimates of smoke specific PM_2.5_. We also chose to be conservative in applying a linear concentration–response function because other studies have suggested higher slopes at lower PM_2.5_ concentrations (Pope et al. 2009).

In the absence of empirical PM data for many regions most severely affected by LFS, we evaluated our results against global data sets of visibility and ground-based AOD, both of which are proxies for particulate air pollution. Although there was considerable regional variation in the degree of correlation with these independent measures, the estimated PM_2.5_ performed comparatively well in sub-Saharan Africa and Southeast Asia (the two global regions with highest mortality contributions). Further reductions in uncertainty of the daily exposures could be achieved with the use of higher temporal resolution fire emission inventories. For example, Mu et al. (2011) used active fire observations from *Aqua*, *Terra*, and *GOES* satellites to develop a daily and 3-hourly fire emissions product for the 2002–2010 period.

The WHO subregions with the highest mortality were those we identified as being chronically affected by LFS (Figure 2). The principal estimate of 339,000 annual deaths is composed of 81% mortality due to chronic exposure and 19% due to sporadic exposure. When the analysis was run under the sporadic-only and chronic-only assumptions (Table 2), WHO subregions identified as chronically affected contributed 53% of the total estimates (138,000 and 520,000, respectively) in both cases.

Previous estimates of the global mortality associated with urban air pollution (Cohen et al. 2005) and smoke from household solid fuel use (Lopez et al. 2006b) assumed purely chronic exposure to PM. Our distinction between chronic and sporadic impacts is a departure from this approach, reflecting the current state of epidemiological evidence and the nature of LFS exposure. On the one hand, only a few studies have reported on the mortality effects of LFS (Hänninen et al. 2009; Morgan et al. 2010; Sastry 2002), and all have estimated associations with short-term fluctuations in PM concentrations. On the other hand, urban air pollution studies have clearly demonstrated that chronic exposure to PM is associated with greater increases in mortality than are short-term fluctuations (Pope and Dockery 2006). LFS is episodic in many parts of the world, and annual average exposures are not appropriate for estimating smoke-related mortality in those regions. Similarly, fire smoke exposure is more chronic (because of high seasonal averages) in some regions, and mortality estimates based on short-term fluctuations might be overly conservative. To date, the short-term mortality impacts for PM from landscape fires have been consistent with those of urban PM. Thus, we considered it reasonable to estimate the chronic effects of PM from LFS using conservative values for the chronic effects of PM from urban sources until more specific evidence becomes available. We were also unable to account for different population responses to air pollution. Although our coefficient for acute exposure was driven by a study in Southeast Asia, no studies conducted in sub-Saharan Africa were available.

Estimates of counterfactual exposures are highly uncertain. Human influence on landscape fire activity varies considerably between ecoclimatic regions. We set the theoretical minimum for PM_2.5_ from LFS as the lowest estimated for each chronically affected WHO subregion over the decade-long study period. However, variation in fire activity during the last decade will not necessarily capture the reduction in fire activity that could be achieved in each environment. For example, tropical rainforests and peat swamps, the primary source of fire emissions in Southeast Asia, rarely burn without human instigation. If such deforestation fires were to be halted, fire activity in this region (and the associated mortality) would be minimal. However, the role of human fire management in savannas, the primary source of emissions in Africa, is less well understood because fire is an integral part of these landscapes (van der Werf et al. 2008). The large estimated influence of El Niño on mortality related to LFS implies that the burden may change in the future if climate change modifies the El Niño Southern Oscillation or drier conditions occur in places with adequate fuels and ignition sources.

Landscape fire activity has been recognized as a global-scale environmental challenge because plumes transgress international boundaries and component gases and particles contribute to climate change (Bowman et al. 2009; Pope and Dockery 2006; van der Werf et al. 2008). This first attempt to quantify the global burden of mortality attributable to LFS has demonstrated important impacts at regional and global scales. We anticipate that subsequent estimates will be improved by better exposure assessment (particularly as empirical PM data become more globally available), further epidemiological studies on mortality and morbidity associated with LFS (particularly in regions with high exposure), and improved understanding of how fire regimes can be modified to reduce smoke emissions. Reducing population level exposure to air pollution from landscape fires is a worthwhile endeavor that is likely to have immediate and measurable health benefits. Such interventions could also potentially provide benefits for the mitigation of climate change and slowing the loss of biodiversity.

## Supplemental Material

(2 MB) PDF
