# Supplementary material for: Estimated Global Mortality Attributable to Smoke from Landscape Fires
Source: Environ Health Perspect. 2012 Feb 18;120(5):695–701. doi: 10.1289/ehp.1104422 (PMC3346787; doi:10.1289/ehp.1104422)
Supplement: (2 MB) PDF [file ehp.1104422.s001.pdf]

Supplemental Material.

Estimated global mortality attributable to smoke from landscape fires

Fay H. Johnston[1], Sarah B. Henderson[2,3,] Yang Chen[4], James T. Randerson[4], Miriam Marlier[5], Ruth S. DeFries[6], Patrick Kinney[7], David M.J.S. Bowman[8] and Michael Brauer[9],

1. Menzies Research Institute, University of Tasmania, Australia

2. University of Tasmania, Australia

3. British Columbia Centre for Disease Control, Canada.

4. Department of Earth System Science, University of California, Irvine, USA

5. Department of Earth and Environmental Sciences, Columbia University, USA

6. Ecology, Evolution, and Environmental Biology, Columbia University, USA

7. Mailman School of Public Health, Columbia University, USA

8. School of Plant Science, University of Tasmania, Australia

9. The University of British Columbia, Canada

Correspondence to fay.johnston@utas.edu.au

Contents

1. Overview 3

2. Model simulations of surface $PM_{2.5}$ and AOD 3

3. Using satellite column AOD data to optimize simulated surface PM2.5 4

Figure 1. Spatial locations of the 14 terrestrial GFED regions. 6

Table 1. Total fire emissions of the sum of organic and black carbon aerosols 7

Figure 2. Six year (2001-2006) mean monthly modeled AOD without (GC) and with satellite constraints (GC-MODIS and GC-MISR) for fire-affected areas in each GFED region 8

4. Evaluation 9

4.1 Evaluation using surface measurements of aerosol optical depth 9

Figure 3. Comparison of estimated AOD and AERONET AOD 10

Figure 4. Comparison of estimated AOD and AERONET AOD in fire affected regions 11

4.2 Evaulation using surface $PM_{2.5}$ observations 12

Figure 5. Comparison of estimated $PM_{2.5}$ and IMPROVE $PM_{2.5}$ 13

Table 2. Global fire aerosol emissions from literature and derived in study averaged over 1997-2006. 14

4.3 Evaluation using surface visibility observations 15

Figure 6. Correlation between visibility and $PM_{2.5}$ estimates 15

5. The distribution of final estimates 16

Figure 7. Distribution of estimates of the global burden of mortality from landscape fire smoke 1997-2006. 16

6. References 17

1. Overview

This paper describes the methods we used for estimating $PM_{2.5}$ from global fires and the approaches used for evaluation. Our estimation of surface level concentrations of particulate matter of less than 2.5 µm ($PM_{2.5}$) from landscape fires involved the following steps: (1) running atmospheric forward model aerosol simulations and estimating surface $PM_{2.5}$ and column aerosol optical depth (AOD); (2) using satellite column AOD data to scale the model estimates of surface $PM_{2.5}$; and (3) evaluating our approach using surface AOD, $PM_{2.5}$, and visibility observations.

2. Model simulations of surface $PM_{2.5}$ and AOD

We used a global 3-D chemical transport model (GEOS-Chem) to simulate the transport, transformation, and deposition of aerosol particles emitted from wildfires and other sources (Bey et al. 2001). The model was driven by assimilated meteorological observations from the Goddard Earth Observing System (GEOS) of the NASA Global Modeling and Assimilation Office (GMAO). In this study we used version v8-01-01 of the model and GEOS-4 meteorology with a 2° (latitude) × 2.5° (longitude) horizontal resolution and 30 vertical layers between the surface and 0.01 hPa.

We performed two sets of full-chemistry (NO_x-O_x-hydrocarbon-aerosol) simulations over a 10-year period (1997-2006). The first simulation included all emission sources of aerosols and aerosol precursors (fossil fuel, biofuel, biomass burning, dust, and sea salt formation). The monthly-resolved fire emissions were based on the Global Fire Emission Database (GFED) version 2 (van der Werf et al. 2006), which combines satellite observations of burned area (Giglio et al. 2006), with estimates of fuel loads obtained from a biogeochemical model driven by other satellite data. Different emission factors of organic carbon (OC) and black carbon (BC) were assumed for fires in tropical forests, savannas, and extra-tropical forests based on the mean of field studies

as reported in Andreae and Merlet (2001). In the second simulation, we excluded GFEDv2 emissions in order to separate the contribution from wildfires.

In our model simulations, we recorded monthly mean 3-D concentrations of different aerosol species: sulfate, OC, BC, sea salt (accumulation mode), sea salt (coarse mode), and dust (7 size bins). We assumed lognormal size distributions for each aerosol species. The modal radii, geometric standard deviation, and density of particles, as well as their hygroscopic growth factors as a function of relative humidity (RH) were from Chin et al (2002). The modeled $PM_{2.5}$ values for each aerosol species were calculated using monthly mean surface concentrations and RH-determined size distribution. Fire $PM_{2.5}$ (denoted as $PM25_{GC\text{-}fire}$) was the difference between the total $PM_{2.5}$ ($PM25_{GC}$) from the two simulations with and without fire emissions. We initialized the model by running GEOS-Chem for July-December in 1996, using all-year (1997-2006) mean monthly emissions from GFEDv2. The two simulations then covered the 1997-2006 period, in which the results were used to derive our exposure estimates. We also converted the mass concentration in each grid cell to AOD for different aerosol species which were assumed to be externally mixed (Tegen and Lacis 1996). We estimated monthly mean column AODs (at 550nm wavelength) for total aerosols (AOD_{GC}) and for fire aerosols ($AOD_{GC\text{-}fire}$).

The $PM_{2.5}$ concentrations from fires derived from the difference between the two simulations described above was our forward model estimate. Given uncertainties in BC and OC emissions and emission factors for these species, we also performed two optimizations of the emissions using observations of AOD from two satellites. Our approach for the optimization is described below.

3. Using satellite column AOD data to optimize simulated surface $PM_{2.5}$

Satellite remote sensing is a reliable way to record continuous information about spatial and temporal distributions of global aerosols (King et al. 1999). AOD determined from satellite remote sensing has been used to derive ground-level $PM_{2.5}$ in previous studies (e.g., Liu et al. 2004; van Donkelaar et al. 2006, 2010). By

taking a similar approach, here we used AOD observations from two satellite instruments to optimize our estimates of surface $PM_{2.5}$, making scalar adjustments for 14 different continental scale regions. MODerate resolution Imaging Spectroradiometer (MODIS) (Remer et al. 2005) and Multi-angle Imaging SpectroRadiometer (MISR) (Martonchik et al. 2009), aboard the NASA Terra satellite have been retrieving AOD under cloud free conditions since 2001 (Chin et al. 2009b). Monthly level 3 AOD data (collection 5 MOD08_M3 and version 22 MISR_AM1_CGAS) at mid-visible wavelengths were downloaded from the NASA Goddard Space Flight Center's Atmosphere Archive and Distribution System (http://ladsweb.nascom.nasa.gov) and the NASA Langley Research Center's Atmospheric Sciences Data Center (http://eosweb.larc.nasa.gov), respectively. We re-gridded the original MODIS (1°×1°) and MISR (0.5°×0.5°) L3 data into the 2°×2.5° resolution of our atmospheric model.

In our approach, we scaled the simulated levels of atmospheric aerosols to match satellite observations of AOD for the period of 2001-2006 using the forward model AOD and satellite-observed AODs (AOD_{MODIS} and AOD_{MISR}). Unlike previous studies that simply used a single scaling factor for all aerosols, we estimated two time-invariant scaling factors for fire aerosols (α) and all other aerosols (β) for the 14 different geographic regions of the GFED, maintaining the same seasonal and regional patterns of aerosol distributions as predicted by the forward model simulations described above. This scaling approach implicitly assumed that the chemical composition of the fire-emitted aerosol (including the ratio of BC to OC in $PM_{2.5}$) remained the same as in the GEOS-Chem simulation described in Supplemental Material Section 2.

The scaling factors were derived using satellite and modeled AODs in fire-affected area, these being defined as the top 1/3 area with highest $AOD_{GC\text{-}fire}$ in each region (Figure S1). For each fire-affected area, we also defined the four months of a mean annual cycle with lowest ratio of $AOD_{GC\text{-}fire}$ to AOD_{GC} (averaged over 2001-2006) as non-fire months. Other sources often dominated the total aerosol distribution during the non-fire months and so we used these months to derive β. β was calculated so that the mean value of scaled AOD_{GC} ($\beta\times AOD_{GC\text{-}other}+AOD_{GC\text{-}fire}$) in the non-fire months was equal to the satellite observed AOD for the same period. Using the

derived β, we then derived α so that the adjusted AOD ($\alpha \times AOD_{GC\text{-}fire} + \beta \times AOD_{GC\text{-}other}$) in the fire months (the four months of a mean annual cycle with highest fire contributions during 2001-2006) matched the satellite observations. Two sets of scaling factors α and β were estimated from MODIS and MISR observations, individually, and are shown in Table S1. Figure S2 shows the monthly AODs for each fire-affected area before and after applying the scaling factors. The derived scaling factors (α and β) for each GFED region were applied to modeled surface $PM_{2.5}$ in all grid cells and all months to derive the satellite optimized datasets: $PM25_{GC\text{-}sat} = \alpha \times PM25_{GC\text{-}fire} + \beta \times PM25_{GC\text{-}other}$ where 'sat' can be either MODIS or MISR.

Our best estimate of surface $PM_{2.5}$ from LFS ($PM25_{mean\text{-}fire}$) combined information from both the forward model and the two satellite-optimized estimates (with double weight on the model): $PM25_{mean\text{-}fire} = (2*PM25_{GC\text{-}fire} + PM25_{GC\text{-}MODIS\text{-}fire} + PM25_{GC\text{-}MISR\text{-}fire})/4$.

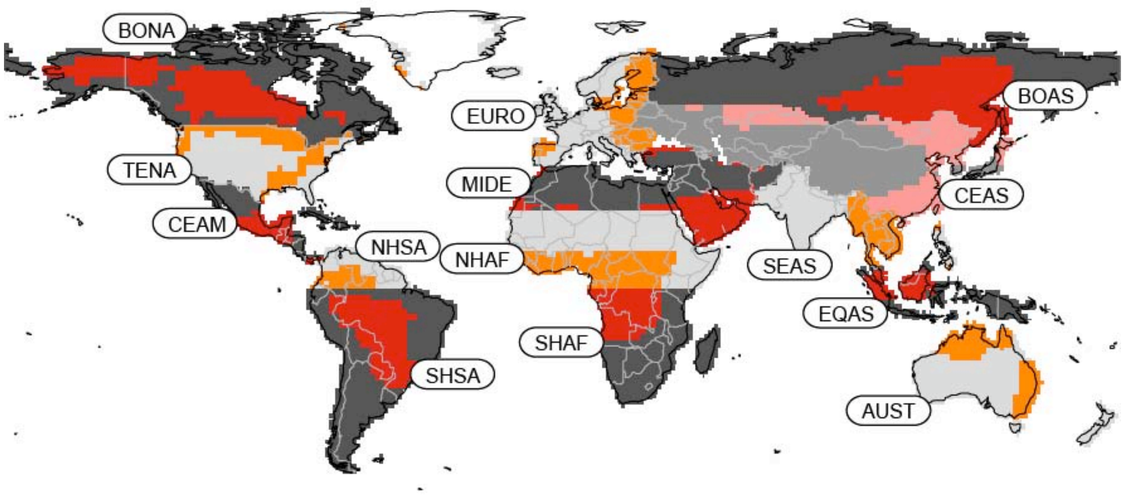

Supplemental Material, Figure 1. Spatial locations of the 14 terrestrial GFED regions used in global fire emissions modeling. The warm colors (red, orange, pink) represent the fire-affected area, which is defined as the top 1/3 area with highest $AOD_{GC\text{-}fire}$ in each GFED region. Satellite AODs and model results in these regions were used to derive scaling factors α and β.

Supplemental Material, Table 1. Total fire emissions of the sum of organic and black carbon aerosols. The units of emissions (E) are Tg yr^{-1}. These emissions are averaged over 1997-2006 in each GFED[1] region and are provided for the original model[2] ($E_{GC\text{-}fire}$), the estimates constrained by MODIS ($E_{GC\text{-}MODIS\text{-}fire}$) or MISR ($E_{GC\text{-}MISR\text{-}fire}$), and our best estimates ($E_{GC\text{-}mean\text{-}fire}$). α and β are unitless scaling factors for fire aerosols and other aerosols, respectively.

**Region**[3]	$E_{GC\text{-}fire}$	$E_{GC\text{-}MODIS\text{-}fire}$			$E_{GC\text{-}MISR\text{-}fire}$			$E_{GC\text{-}Mean\text{-}fire}$
		α	β	E	α	β	E	
**BONA**	0.67	1.54	2.08	1.03	2.26	1.03	1.51	0.97
**TENA**	0.25	8.35	1.00	2.07	9.21	0.86	2.28	1.21
**CEAM**	0.65	6.87	2.06	4.45	5.22	2.26	3.38	2.28
**NHSA**	0.40	2.48	2.91	0.98	2.70	3.22	1.07	0.71
**SHSA**	3.05	3.30	1.70	10.1	1.90	2.66	5.79	5.48
**EURO**	0.14	4.56	0.58	0.63	5.54	0.45	0.77	0.42
**MIDE**	0.01	11.1	0.52	0.06	9.36	0.54	0.05	0.03
**NHAF**	4.82	1.08	0.61	5.21	1.02	0.61	4.92	4.94
**SHAF**	4.29	2.01	3.23	8.62	1.68	3.34	7.20	6.10
**BOAS**	2.89	1.16	1.83	3.36	0.86	0.89	2.49	2.91
**CEAS**	0.41	6.03	0.96	2.50	3.16	0.65	1.31	1.16
**SEAS**	1.62	3.08	1.77	5.00	2.43	1.80	3.94	3.05
**EQAS**	3.20	2.72	4.08	8.71	2.30	4.43	7.37	5.62
**AUST**	1.14	2.04	1.33	2.33	3.00	1.69	3.43	2.01
**Global**	23.5			55.0			45.5	36.9

1 Global Fire Emission Database

2 The original model refers to the atmospheric model aerosol simulations with the GEOS-Chem three dimensional chemical transport model.

3See Supplemental Figure 1 for the location of each region. These regions are continental-scale regions used in fire emissions modeling and are defined independently of the WHO regions described in the main text.

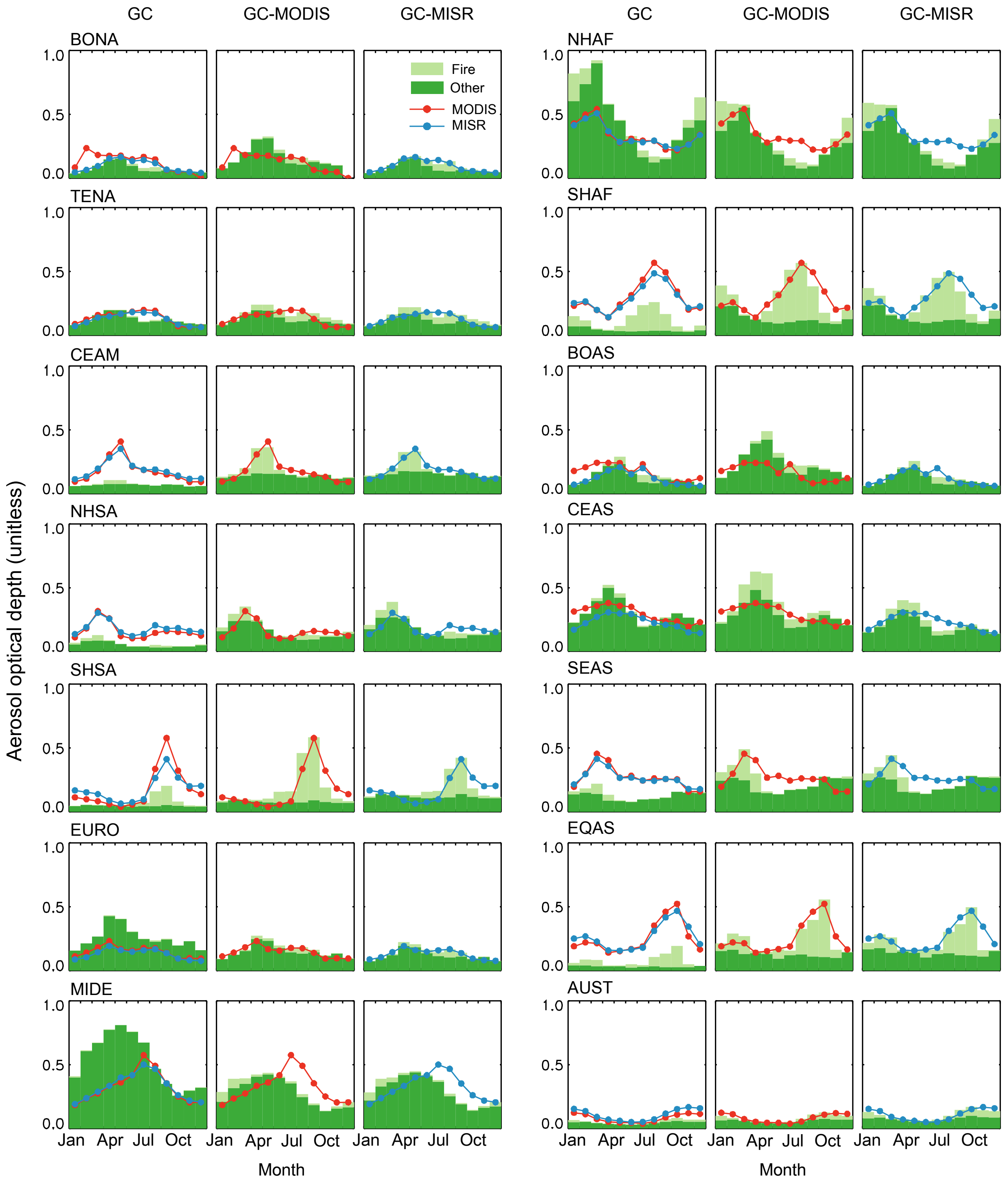

Supplemental Material, Figure 2. Six year (2001-2006) mean monthly modeled AOD without (GC, vertical bars) and with satellite constraints (GC-MODIS and GC-MISR) for fire-affected areas in each GFED region (as denoted by the red, orange and pink areas in Figure S1). The satellite AOD observations for each fire-affect area also are shown in each panel, with MODIS AODs represented by a red line and MISR with a blue line.

4. Evaluation

The method of combining satellite-based AOD and GEOS-Chem simulations to derive surface $PM_{2.5}$ has been evaluated in previous studies (e.g., van Donkelaar et al. 2010). Since the objective of this study is to quantify the health effects of landscape fire smoke, we focus on the evaluation of our exposure estimates in regions where fire is an important source of aerosols. We used (1) ground-based AOD, (2) Surface $PM_{2.5}$, and (3) visibility observations (Holben et al. 1998; NOAA 2009). Visibility and AOD are both directly affected by PM concentrations and provide our best available indication of changes in air quality from all sources in places where PM observations are not available.

4.1 Evaluation using surface measurements of aerosol optical depth

We compared the spatial and temporal variability of our AOD estimates with the observations from the Aerosol Robotic Network (AERONET), (Holben et al. 1998). Figure S3 shows the scatter plots of modeled AOD and AERONET AOD (Level 2.0 cloud-screened, quality-assured, at 500nm wavelength, version 2) in regions where annual mean fire AODs were greater than 0.01. The model results were sampled at the location and time of each observation. Figure S4 compares the best estimate of AOD with AERONET in different continental-scale GFED regions (Figure S1). We found in regions with significant fire emissions, the best estimate AOD, which combined model simulations and satellite constraints, agreed better with the AERONET observations compared to model-only or satellite-only estimates. Our estimated AOD showed strong positive correlation with ground based measurements from the AERONET network in regions with the highest amounts of fire emissions, including Southeast Asia ($r = 0.76$, $n = 148$), Southern Hemisphere Africa ($r = 0.81$, $n = 119$ and Northern Hemisphere Africa ($r = 0.90$, $n = 74$). In the other regions of the world the correlation was more variable ranging from 0.17 in the North American boreal zone to 0.65 in Australia (Figure S4). The mean values from our best estimate AOD were, on average, slightly smaller than the AERONET observations (Figure S3).

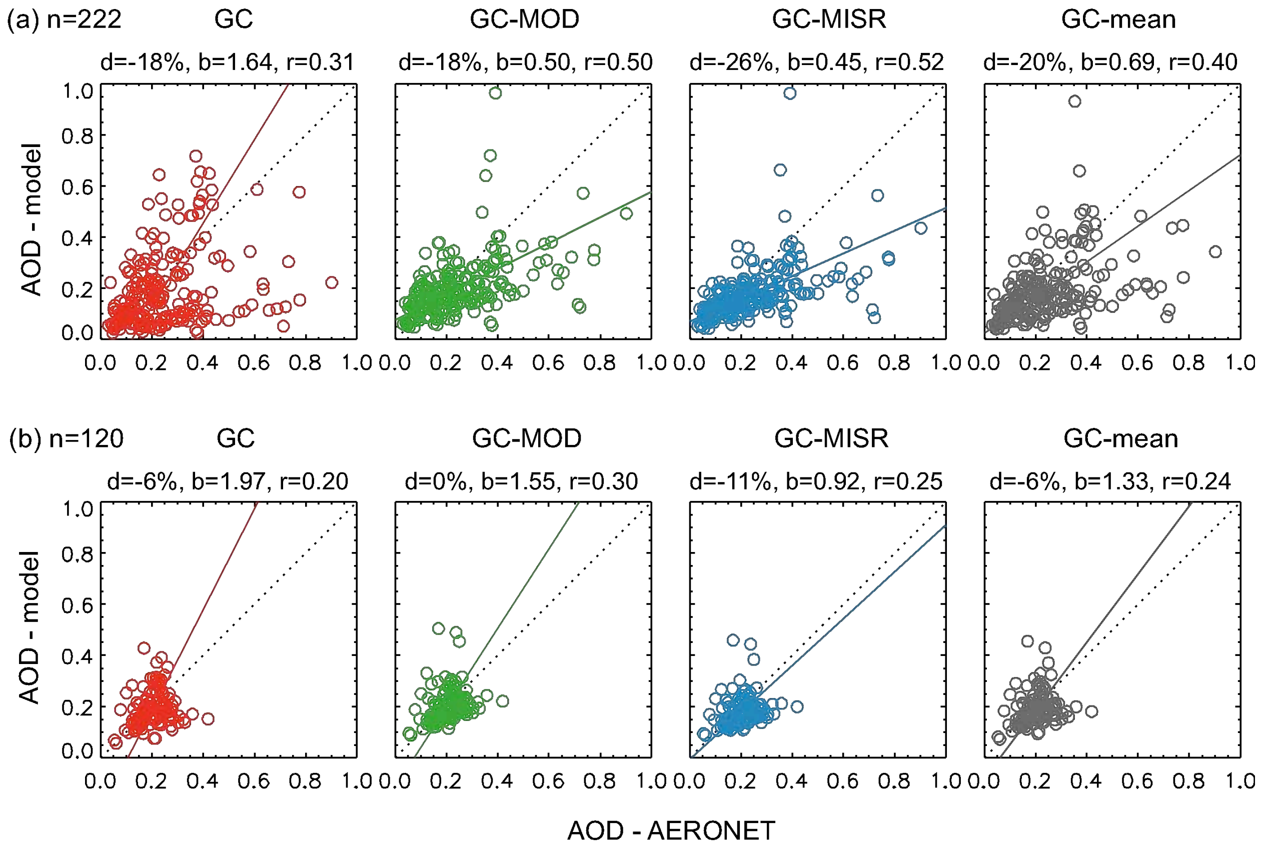

Supplemental Material, Figure 3. Comparison of estimated and AERONET AOD (unitless) for stations in regions where annual AOD from fire emissions was greater than 0.01 as simulated by GEOS-Chem. Each dot in (a) represents the mean AOD at a single site during the period of 1997-2006. Each dot in (b) represents the all-site mean AOD in each month. The statistics of these regressions, including sample number (*n*), mean bias (*d*), slope (*b*), and linear correlation coefficient (*r*) are shown above each panel.

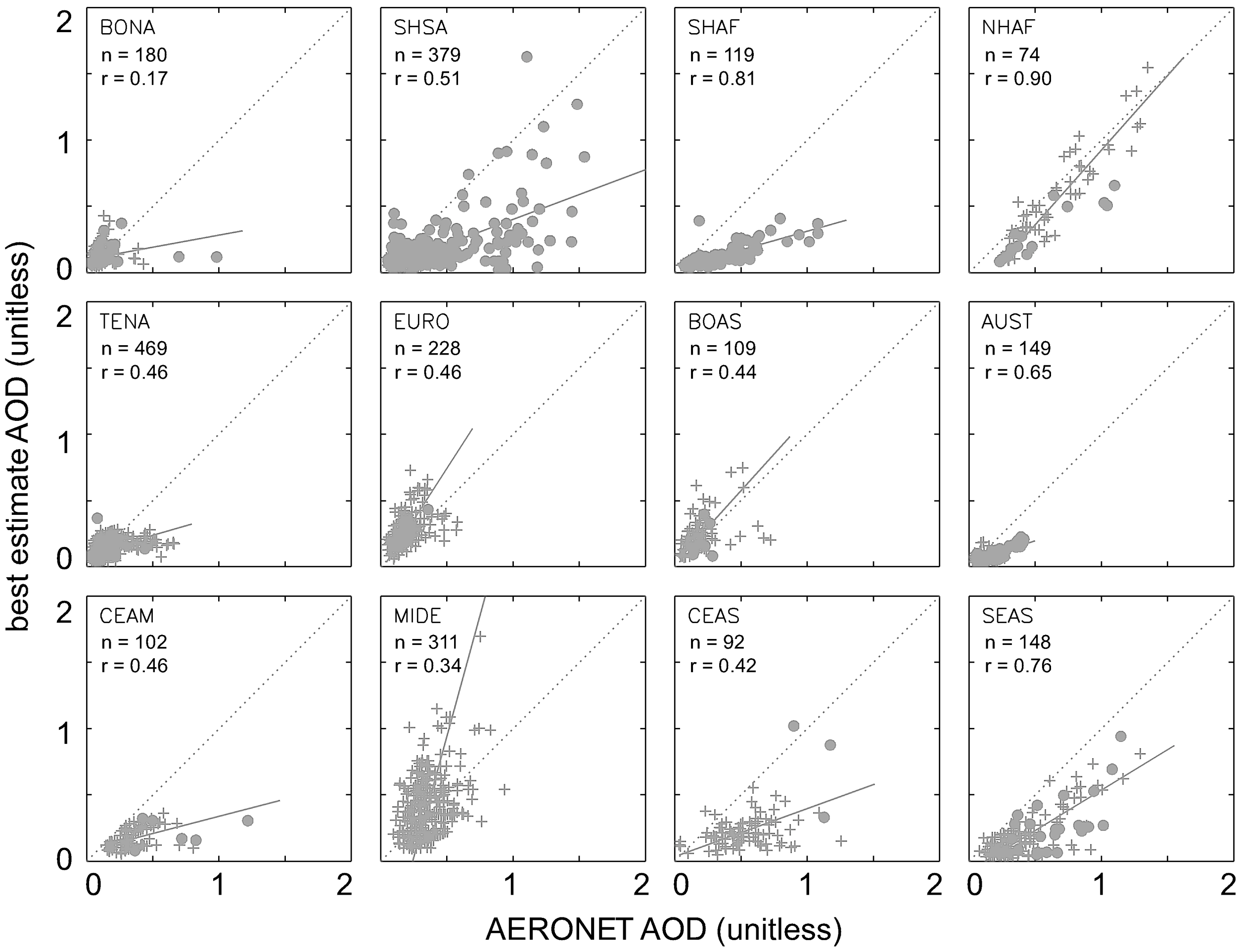

Supplemental Material, Figure 4. Comparison of estimated and AERONET AOD for fire-affected areas of each GFED region. EQAS and NHSA are omitted due to small number of stations. Each dot represents a monthly mean AOD at a single site during the period of 1997-2006. Circles and plus signs are for months in which the fire contribution to total AOD was greater than and less than 20%, respectively.

4.2. Evaluation using surface $PM_{2.5}$ observations

The United States EPA Interagency Monitoring of Protected Visual Environments (IMPROVE) program (Chow and Watson 2002) has been measuring air quality parameters in United States since 1985 (http://vista.cira.colostate.edu/improve/). $PM_{2.5}$ values from IMPROVE after 1999 were available at the Visibility Information Exchange Web System (VIEWS) (http://views.cira.colostate.edu/web/). We compared the monthly all-site mean and site-resolved all-year mean $PM_{2.5}$ in the US from IMPROVE with model results and satellite-constrained model results (Figure S5). Both model estimation and satellite-constrained values agreed well with IMPROVE when the $PM_{2.5}$ concentrations were low (<7μm/m^3). For high aerosol concentrations, the model tended to underestimate the $PM_{2.5}$ while the two satellite-constrained estimates tended to overestimate the observed values. The dataset which combined model and satellites ($PM25_{mean}$) had highest correlation with the IMPROVE observations and showed no significant bias.

A major source of the uncertainty in model simulation of AOD and $PM_{2.5}$ comes from the emission factors (EF) of aerosol species from fires. Recently, *Chin et al.* (2009a) and *Reid et al.* (2009) used EFs for OC and BC that are 40%-100% larger than those derived from earlier field studies (Andreae and Merlet 2001). These adjustments appear to substantially improve model estimates of AOD and $PM_{2.5}$ as compared with satellite and surface network observations. The EFs used in this study were at the lower end of the range in literature (Table S2). In addition, there is usually a gap between the $PM_{2.5}$ emission factors and the sum of BC and OC emission factors e.g., *Andreae and Merlet* (2001) (see Table S2). If we used the difference between the $PM_{2.5}$ EF and BC EF as the EF for particulate organic matter (POM) (the approach taken by *Ito and Penner* (Ito and Penner 2005) and *Dentener et al.* (2006), the GEOS-Chem modeled $PM_{2.5}$ would have been 40% higher. By using the satellite observations as a constraint, the underestimation of model simulations during high fire seasons was reduced to a certain extent. Our best estimate ($PM25_{mean}$) agreed better with satellite and AERONET observations than the original model simulation (Figures S2 and S3).

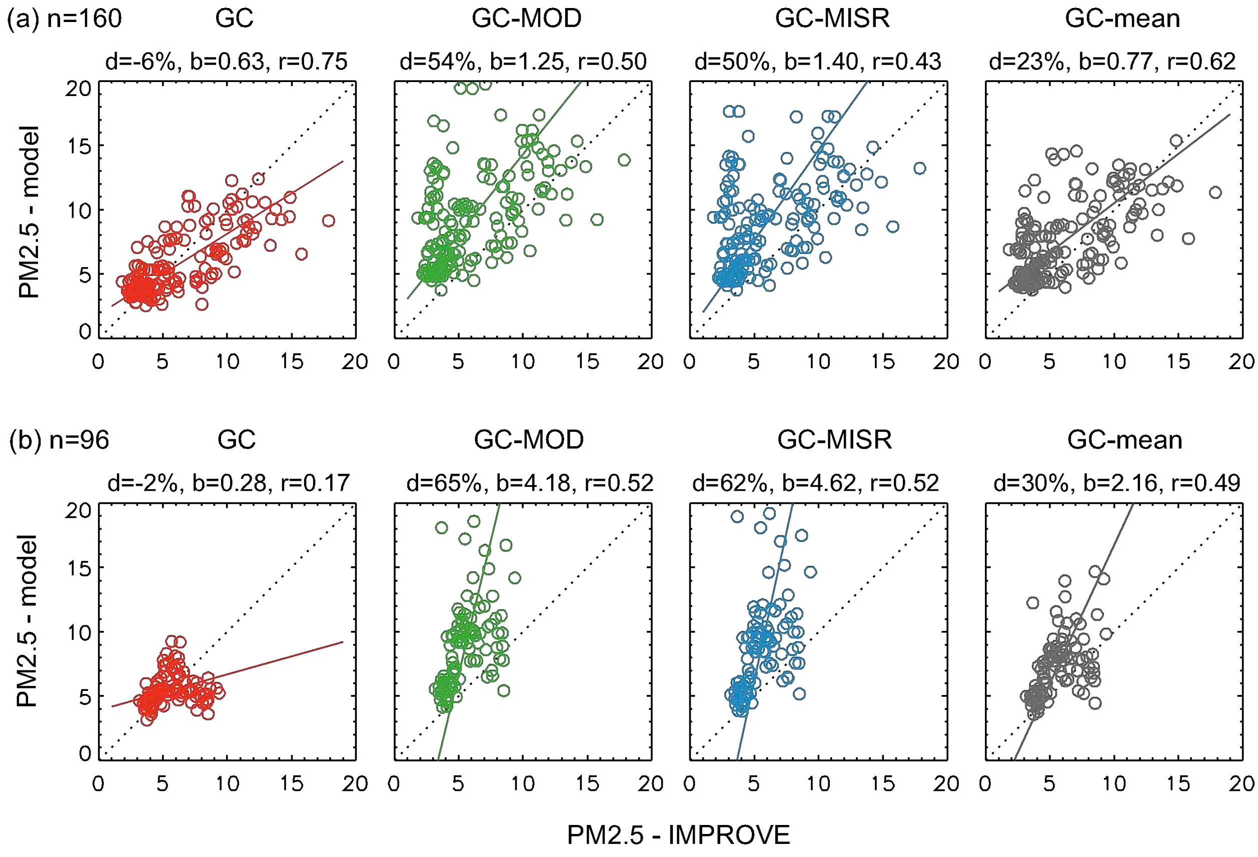

Supplemental Material. Figure 5. Comparison of estimated $PM_{2.5}$ and IMPROVE $PM_{2.5}$ (in μg/m^3) in regions where annual $PM_{2.5}$ from fire source is greater than 1 μg/m^3. Each dot in (a) represents the mean $PM_{2.5}$ at a single site during the period of 1997-2006. Each dot in (b) represents the all-site mean $PM_{2.5}$ in each month. The statistics of these regressions, including sample number (*n*), mean bias (*d*), slope (*b*), and linear correlation coefficient (*r*) are shown above each panel.

Supplemental Material, Table 2. Global fire aerosol emissions from literature and derived in study averaged over 1997-2006[1].

Study	Global emissions (Tg yr^{-1})	Emission factors (EF, g kg^{-1}) used in the reported study and related references	
Andreae and Merlet (2001)	2.7 (BC) + 23.4 (OC)<br>36.4 ($PM_{2.5}$)	Savanna:	0.48 (BC),3.4 (OC),5.4 ($PM_{2.5}$)
		Trop. forest:	0.66 (BC), 5.2 (OC),9.1 ($PM_{2.5}$)
		Extrop. forest:	0.56 (BC), 8.6-9.7 (OC), 13.0 ($PM_{2.5}$)
			Andreae and Merlet (2001)
Penner et al. (2001)	5-9 (BC) + 45-80 (OC)	Based on a review of previous studies	
Chin et al. (2002)	11 (BC) + 77 (OC)	2 (BC), 14 (OC)	
			Chin et al. (2002)
Ito and Penner (2004)	22.1-35.3 ($PM_{2.5}$)	Savanna:	5.4 ($PM_{2.5}$)
		Trop. forest:	9.1 ($PM_{2.5}$)
		Extrop. forest:	13.0 ($PM_{2.5}$)
			Andreae and Merlet (2001)
Bond et al. (2004)	3.3 (BC) + 25.0 (OC)	Savanna:	0.48 (BC), 3.4 (OC)
		Forest:	0.56-0.61 (BC), 5.2-8.0 (OC)
			Bond et al (2004)
Ito and Penner (2005)	3.6 (BC) + 29 (POM)	Savanna:	0.48 (BC), 4.9 (POM)
		Trop. forest:	0.66 (BC), 8.4 (POM)
		Extrop. forest:	0.56 (BC,), 12.4 (POM)
			Andreae and Merlet (2001)
Dentener et al. (2006)	3.1 (BC) + 34.7 (POM)	Savanna:	0.46 (BC), 4.4 (POM)
		Trop. forest:	0.63 (BC), 8.5 (POM)
		Extrop. forest:	0.56 (BC), 12.4 (POM)
			van der Werf et al.(2006)
Chin et al. (2009a)	5 (BC) + 40.2 (OC)	1 (BC), 8 (OC)	
			Chin et al.(2009a)
Reid et al. (2009)	44 ($PM_{2.5}$, based on GFED C emission)<br>110 ($PM_{2.5}$, based on FLAMBE C emission)	Savanna:	7 ($PM_{2.5}$)
		Woody savanna:	8.5 ($PM_{2.5}$)
		Trop. forest :	12 ($PM_{2.5}$)
		Temp. forest:	17 ($PM_{2.5}$)
		Boreal forest:	16 ($PM_{2.5}$)
			Reid et al. (2005)
van der Werf et al. (2006)[2]	2.5 (BC) + 21.0 (OC)	Savanna:	0.46 (BC), 3.2 (OC)
		Trop. forest:	0.63 (BC), 5.2 (OC)
		Extrop. forest:	0.56 (BC), 9.1 (OC)
			van der Werf et al.(2006), Andreae and Merlet (2001)
This study (MODIS optimized)	5.9 (BC) + 49.1 (OC)		
This study (MISR optimized)	4.8 (BC) + 40.7 (OC)		
This study (best estimate)	3.9 (BC) + 33.0 (OC)		

[1] Abbreviations are as follows: BC, black carbon; OC, organic carbon; $PM_{2.5}$, particulate matter < 2.5µm in diameter; POM, particulate organic matter, defined as $PM_{2.5}$ component that does not belongs to BC; GFED, Global Fire Emission Database; FLAMBE, Fire Location and Modeling of Burning Emissions.

[2] Also used in this study for the atmospheric model simulation with GEOS-Chem.

4.3. Evaluation using surface visibility observations

Surface measurements of $PM_{2.5}$ were not readily available for most tropical regions with high fire emissions. We therefore compared our estimates of $PM_{2.5}$ with visibility data in three regions with high fire activity: Southern Hemisphere South America, sub-Saharan Africa, and Southeast Asia. Visibility data are available from the National Climatic Data Center Global Summary of the Day (NOAA 2009), as 24-hour averages recorded to the nearest 0.16 kilometers. We followed the detailed framework presented in Husar *et al.* (2000), in which visibility is converted to the aerosol extinction coefficient (β_{ext} km^{-1}) and the data are filtered to reduce the impact of precipitation and observational bias on measurement quality (Husar et al. 2000). Monthly β_{ext} values were compared to our $PM_{2.5}$ estimates. We extracted estimates from the 2x2.5 degree grid box that contained the station location and computed the linear correlation with the ground measurements. Any stations that were located within the same grid box were averaged. The median correlation coefficients were 0.57 for Africa (n=58), 0.60 for South America (n=47), and 0.68 for Southeast Asia (n=13) (Figure S6).

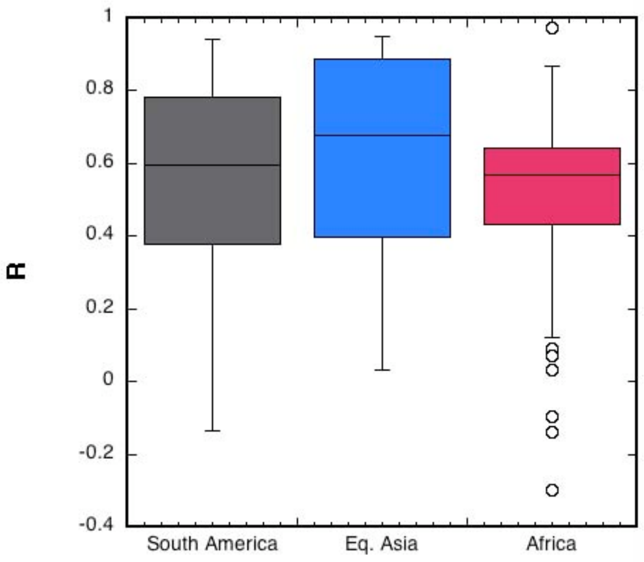

Supplemental Material. Figure 6. Correlation between visibility and $PM_{2.5}$ estimates in South America (n=47), Equatorial Asia (n=13) and Sub-Saharan Africa (n=58).

5. The distribution of final estimates

The figure below displays the outputs produced by all permutations of each plausible assumption concerning landscape fire smoke exposure, counterfactual exposures and concentration response associations as described in Table 2 of the main manuscript. N= 2,192, the median estimate was 379,000 and the inter-quartile range was 260,000 – 600,000. The base-case estimate of 339,000 is shown by the red dashed line.

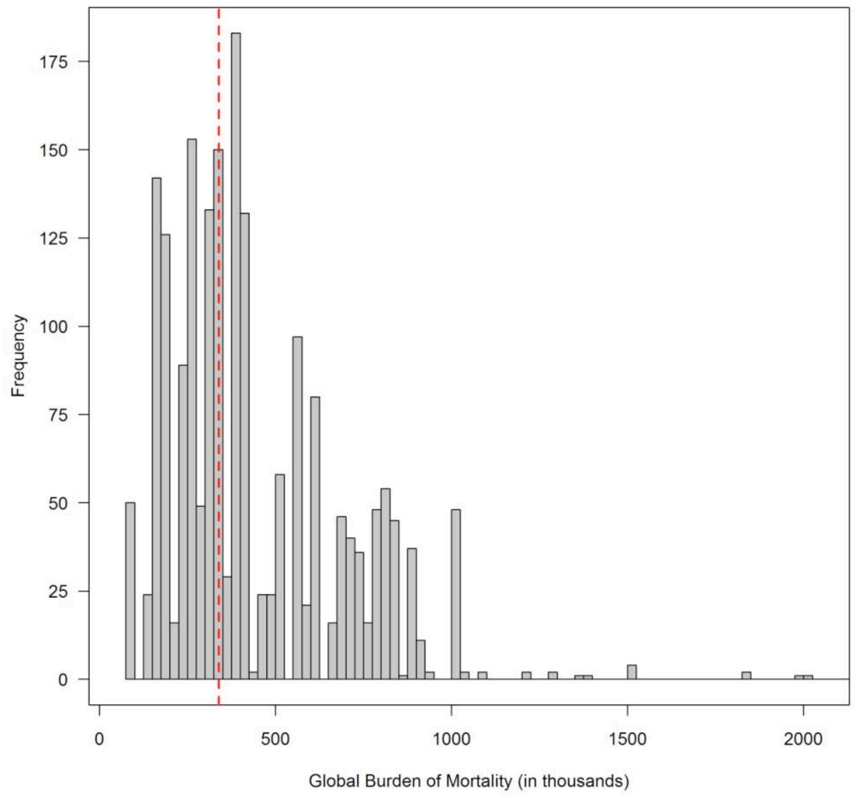

Supplemental Material, Figure 7. Distribution of estimates of the global burden of mortality from landscape fire smoke 1997-2006.

6. References

Andreae MO, Merlet P. 2001. Emission of trace gases and aerosols from biomass burning. Global Biogeochemical Cycles 15(4):955-966.

Bey I, Jacob DJ, Yantosca RM, Logan JA, Field BD, Fiore AM, et al. 2001. Global modeling of tropospheric chemistry with assimilated meteorology: Model description and evaluation. Journal of Geophysical Research-Atmospheres 106(D19):23073-23095.

Bond TC, Streets DG, Yarber KF, Nelson SM, Woo JH, Klimont Z. 2004. A technology-based global inventory of black and organic carbon emissions from combustion. Journal of Geophysical Research 109:D14203.

Chin M, Diehl T, Dubovik O, Eck TF, Holben BN, Sinyuk A, et al. 2009a. Light absorption by pollution, dust, and biomass burning aerosols: a global model study and evaluation with AERONET measurements. Annales Geophysicae 27(9):3439-3464.

Chin M, Ginoux P, Kinne S, Torres O, Holben BN, Duncan BN, et al. 2002. Tropospheric aerosol optical thickness from the GOCART model and comparisons with satellite and Sun photometer measurements. Journal of the Atmospheric Sciences 59(3):461-483.

Chin M, Kahn RA, Remer LA, Yu H, Rind D, Feingold G, et al. 2009b. Atmospheric Aerosol Properties and Climate Impacts. Washington: National Aeronatics and Space Administration.

Chow JC, Watson JG. 2002. $PM_{2.5}$ carbonate concentrations at regionally representative Interagency Monitoring of Protected Visual Environment sites. Journal of Geophysical Research-Atmospheres 107(D21):8344.

Dentener F, Kinne S, Bond T, Boucher O, Cofala J, Generoso S, et al. 2006. Emissions of primary aerosol and precursor gases in the years 2000 and 1750 prescribed data-sets for AeroCom. Atmospheric Chemistry and Physics 6:4321-4344.

Giglio L, van der Werf GR, Randerson JT, Collatz GJ, Kasibhatla P. 2006. Global estimation of burned area using MODIS active fire observations. Atmospheric Chemistry and Physics 6:957-974.

Holben BN, Eck TF, Slutsker I, Tanre D, Buis JP, Setzer A, et al. 1998. AERONET - A federated instrument network and data archive for aerosol characterization. Remote Sens Environ 66(1):1-16.

Husar RB, Husar JD, Martin L. 2000. Distribution of continental surface aerosol extinction based on visual range data. Atmospheric Environment 34(29-30):5067-5078.

Ito A, Penner JE. 2004. Global estimates of biomass burning emissions based on satellite imagery for the year 2000. Journal of Geophysical Research 109:D14S05.

Ito A, Penner JE. 2005. Historical emissions of carbonaceous aerosols from biomass and fossil fuel burning for the period 1870–2000. Global Biogeochemical Cycles 19(2):GB2028.

King MD, Kaufman YJ, Tanre D, Nakajima T. 1999. Remote sensing of tropospheric aerosols from space: Past, present, and future. Bulletin of the American Meteorological Society 80(11): 2229-2259.

Martonchik JV, Kahn RA, Diner DJ. 2009. Retrieval of aerosol properties over land using MISR observations. In: Satellite Aerosol Remote Sensing Over Land, (Kokhanovsky AA, de Leeuw G, eds). Berlin Springer, 267-293.

NOAA. 2009. National Climatic Data Center. Available at www.ncdc.noaa.gov/oa/ncdc.html Date accessed 17 May 2011.

Penner JE, Andreae MO, Annegarn H, Barrie L, Feichter J, Hegg D, et al. 2001. Aerosols, their direct and indirect effects. In: Climate Change 2001: The Scientific Basis: Contribution of Working Group I to the Third Assessment Report of the Intergovernmental Panel on Climate Change, (Houghton JT, Ding Y, Griggs DJ, eds). New York:Cambridge Univ. Press, 291-336.

Reid JS, Eck TF, Christopher SA, Koppmann R, Dubovik O, Eleuterio DP, et al. 2005. A review of biomass burning emissions part III: intensive optical properties of biomass burning particles. Atmospheric Chemistry and Physics 5:827-849.

Reid JS, Hyer EJ, Prins EM, Westphal DL, Zhang JL, Wang J, et al. 2009. Global Monitoring and Forecasting of Biomass-Burning Smoke: Description of and Lessons From the Fire Locating and Modeling of Burning Emissions (FLAMBE) Program. Ieee Journal of Selected Topics in Applied Earth Observations and Remote Sensing 2(3):144-162.

Remer LA, Kaufman YJ, Tanré D, Mattoo S, Chu DA, Martins JV, et al. 2005. The MODIS Aerosol Algorithm, Products, and Validation. Journal of the Atmospheric Sciences 62: 947-973.

Tegen I, Lacis AA. 1996. Modeling of particle size distribution and its influence on the radiative properties of mineral dust aerosol. Journal of Geophysical Research-Atmospheres 101(D14):19237-19244.

van der Werf GR, Randerson JT, Giglio L, Collatz GJ, Kasibhatla PS, Arellano AF. 2006. Interannual variability in global biomass burning emissions from 1997 to 2004. Atmospheric Chemistry and Physics 6:3423-3441.

van der Werf GR, Randerson JT, Giglio L, Collatz GJ, Mu M, Kasibhatla PS, et al. 2010. Global fire emissions and the contribution of deforestation, savanna, forest, agricultural, and peat fires (1997-2009). Atmospheric Chemistry and Physics 10: 11707-11735.

van Donkelaar A, Martin RV, Brauer M, Kahn R, Levy R, Verduzco C, et al. 2010. Global estimates of ambient fine particulate matter concentrations from satellite-based aerosol optical depth: development and application. Environmental Health Perspectives 118(6):847-855.
